# The Hahnemann Monument

**Published:** 1897-11

**Authors:** Joseph T. Cook, A. R. Wright, Wm. C. Letchworth, Anna H. Frost

**Affiliations:** President, 636 Delaware Avenue; Buffalo, N. Y.; Treasurer, 414 Elmwood Avenue; Buffalo, N. Y.; Secretary, 98 Anderson Place; Buffalo, N. Y.; Assistant Secretary, 157 North Pearl Street; Buffalo, N. Y.


### THE HAHNEMANN MONUMENT.

BUFFALO, N. Y., October 28th, 1897.

TO THE HOMŒOPATHIC PHYSICIANS OF THE UNITED STATES:

The Ladies' Hahnemann Monument Association was formed immediately after the meeting of the American Institute of Homœopathy in this city, at the request of several members.

Our reason for existing is to raise money wherever Homœopathy is recognized in this country, for the completion and erection of the artistic monument to the memory of Samuel Hahnemann, now in the sculptor's hands.

The committee fully realize the responsibility and the immense amount of work devolving upon them to make the movement a success. In addition to the enthusiasm felt for a cause which involves a principle as sacred as it is scientific, the women of Buffalo have been induced to supplement the work already inaugurated by the homœopathic physicians of our land, by the earnest hope that every practitioner of this school of medicine will give his or her hearty support for the speedy accomplishment of this great work. You are cordially requested to assist, especially in three ways:

First—To send us the name of one or more competent and influential ladies in your city, one of whom will be appointed chairman of a local committee, to raise money in said city or town.

Second—To encourage your patrons to contribute money, however small the amount may be, to this fund.

Third—To contribute without delay to your local fund, if you have not already given to this object; thus setting the example of prompt giving, which will prove beyond a doubt that you appreciate the value and importance of the efforts put forth by the women of the United States for the glory of Homœopathy.

We beg that you will consider favorably these suggestions, for in no other way can we succeed.

We beg to remain,

Yours very truly,

Mrs. Joseph T. Cook, *President,*
636 Delaware Avenue.

Mrs. A. R. Wright, *Treasurer,*
414 Elmwood Avenue.

Mrs. Wm. C. Letchworth, *Secretary,*
98 Anderson Place.

Miss Anna H. Frost, *Assistant Secretary,*
157 North Pearl Street.

---

A correspondent furnishes us with the particulars of another antitoxin death. The patient, a Miss Florence Beckwith, of Mt. Pleasant, Ill., had been ill for a few hours only. The attending physician pronounced the trouble diphtheria, which had been quite prevalent recently. With the consent of both patient and parents it was decided to employ antitoxin. The usual injection was made on the side of the body, and almost immediately followed by a suffused redness, with itching sensations like nettle rash over the body. Both head and throat pained her, followed by a choking sensation, feeble pulse, and death in five minutes.—*Homœo Envoy.*

---

Apostrophe to Jenner.—A distinguished German physician not long ago thus apostrophized the English inventor of vaccination: "Thou English Doctor Jenner, thou hast brought the laws of Nature into confusion; thou hast made the people ailing; thou hast killed unnumbered innocent children; receive the Devil's thanks from German soil."—*Fashions of the Day in Medicine and Science, by H. Strickland Constable. Leng & Co., Kingston-upon-Hull, 1879, page 40.*

